# Synthesis of prenylated flavonols and their potents as estrogen receptor modulator

**DOI:** 10.1038/s41598-017-12640-9

**Published:** 2017-09-29

**Authors:** Zhenru Tao, Juan Liu, Yueming Jiang, Liang Gong, Bao Yang

**Affiliations:** 1 Key Laboratory of Plant Resources Conservation and Sustainable Utilization, Guangdong Provincial Key Laboratory of Applied Botany, South China Botanical Garden, Chinese Academy of Sciences, Guangzhou, 510650 China; 2 University of Chinese Academy of Sciences, Beijing, 100049 China

## Abstract

Prenylated flavonols are known as phytoestrogen and have good bioactivties. However, their abundances in nature are pretty low. It is required to find an efficient synthesis technique. Icariin is a prenylated flavonol glycoside with low cost. It can be used to synthesize different prenylated flavonols. A combination of cellulase and trifluoacetic acid hydrolysis could effectively remove rhamnose and glucose from icariin. Icaritin, anhydroicaritin and wushanicaritin were the leading prenylated flavonol products. Their affinities to estrogen receptors α and β were predicted by docking study. The weak affinity of wushanicaritin indicated that prenyl hydroxylation impaired its affinity to estrogen receptor β. The prenyl cyclization led to a loss of affinity to both receptors. The interactions between icaritin and ligand binding cavity of estrogen receptor β were simulated. π-π stacking and hydrophobic forces were predicted to be the dominant interactions positioning icaritin, which induced the helix (H12) forming an activated conformation.

## Introduction

Prenylated flavonoids are an important sub-class of flavonoids, which combine a flavonoid skeleton with a lipophilic prenyl side chain^1^. Even though flavonoids are widely present at a high level in nature^2^, the levels of prenylated flavonoids are usually much lower. Prenylated flavonoids have been documented to be more bioactive than their flavonoid precursors, as prenylation increases the lipophilicity of flavonoids, leading to a higher permeability and bioavailability *in vivo* ^3,4^. For instance, 8-prenyl quercetin shows a higher cellular uptake and a lower efflux than quercetin in Caco-2 and C2C12 myotube cells. 8-Prenyl substitution also enhances the bioavailability of quercetin in different tissues *in vivo* ^5^. Prenylated flavonoids possess a wide variety of bioactivities, such as estrogenic activity, immunomodulatory activity, anticancer activity and antioxidant activity^6,7^.

Icaritin is a monoprenylated flavonol with 4′-methoxyl. It has been documented to have osteoblastic and neuroprotective activities^8,9^. It can reduce the incidence of steroid-associated oesteonecrosis in rabbit with inhibition of both intravascular thrombosis and extravascular lipid deposition for maintaining the integrity of intraosseous vasculature^10^. The anti-breast cancer activity of icaritin remains confused. Icaritin at 5 μM can inhibit the proliferation of breast cancer stem/progenitor cells even better than tamoxifen^11^. However, a proliferation stimulatory effect has been observed for icaritin and desmethylicaritin in MCF-7 cells^12^. Moreover, icaritin shows anti-inflammatory activity and inhibitory activities against other cancer cells^13^.

There are two classes of estrogen receptors, α and β. They are expressed in different tissues. Estrogen receptor α regulates the proliferation and differentiation of breast cancer cells. However, estrogen receptor β antagonizes the activities of estrogen receptor α, inhibiting carcinogenesis and tumor progression^14^. Estrogen receptor β expression is positively correlated with an improved clinical response to therapy and its loss is considered as an important step of cancer progression^15^. Therefore, it is of interest to find a selective estrogen receptor modulatory agent. As prenylated flavonols are potent phytoestrogens, it is worthy to find a selective estrogen receptor modulatory agents from this class of chemicals. However, due to their limited abundances in nature, it is required to develop an efficient synthesis technique. Though *Epimedium* genus is a good source of icaritin derivatives, the icaritin level is very low when determined by HPLC-MS^16^. As icariin is abundant in plants of *Epimedium* genus, using it as initial substrate to synthesize icaritin and its derivatives can be a possible way.

## Experimental section

### Chemicals

Icariin, β-dextranase, β-glucosidase were purchased from Solarbio (Beijing, China). Cellulase was obtained from Yakult pharmaceutical Industry Co., LTD (Tokyo, Japan). HPLC grade acetonitrile was purchased from ANPEL Laboratory Technologies Inc. (Shanghai, China).

### Synthesis of icaritin, anhydroicaritin and wushanicaritin

#### Enzyme hydrolysis to remove glucoside

Icariin (100 mg) were added into 25 ml of water. Five milligrams of enzymes were added to start the hydrolysis reaction at 37 °C for 2.5 h. The reaction products were extracted by acetyl acetate. The extract was dried by a vacuum rotary evaporator and redissolved in methanol. The products were qualified and quantified by ultra-performance liquid chromatography.

The effects of temperature, time and enzyme type were investigated. Three enzymes, including β-dextranase, β-glucosidase and cellulase, were tested to compare their hydrolysis efficiencies. When analysing the effect of temperature, 20, 37, 60 and 80 °C were used. The time were set as 1.5, 2.5, 3.5 and 4.5 h, respectively.

#### Acid hydrolysis to remove rhamnoside

Icariin (100 mg) were suspended in 25 ml of water. Trifluoacetic acid (TFA) was added to a final concentation of 2 M. The hydrolysis was conducted at 60 °C for 1 h. The reaction products were extracted by acetyl acetate. The extract was dried by vacuum rotary evaporator and redissolved in methanol. The products were qualified and quantified by ultra-performance liquid chromatography.

#### Combination of cellulase and TFA hydrolysis

Hydrolysis by cellulase first and then TFA: Icariin (100 mg) were added into 25 ml of water. Five milligrams of cellulase were added to start the hydrolysis reaction at 37 °C for 2.5 h. Then TFA was added to a final concentation of 2 M. The hydrolysis was conducted at 60 °C for 1 h. The reaction products were extracted by acetyl acetate. The extract was dried by vacuum rotary evaporator and redissolved in methanol. The products were qualified and quantified by ultra-performance liquid chromatography.

Hydrolysis by TFA first and then cellulose: Icariin (100 mg) were added into 25 ml of water. TFA was added to a final concentation of 2 M. The hydrolysis was conducted at 60 °C for 1 h. The hydrolysates were dried by vacuum rotary evaporator. Deionized water was added and dried again. This step was repeated for four times to completely remove acid. The dry products were redissolved in 25 ml of water. Five milligrams of cellulase were added to start the hydrolysis at 37 °C for 2.5 h. The reaction products were extracted by acetyl acetate. The extract was dried by vacuum rotary evaporator and redissolved in methanol. The products were qualified and quantified by ultra-performance liquid chromatography.

### Preparation and identification of the reaction products

The enzymatic products were purified by semi-preparative reversed-phase liquid chromatography. 1.5 g of reaction products were loaded into a column with 15 × 460 mm. Methanol and water were used for elution. The column was equilibrated at 10% methanol for 12 min; 12–52 min, 75% methanol; 52–92 min, 80% methanol; 92–132 min, 85% methanol; 132–172 min, 90% methanol; 172–212 min, 100% methanol. The flow rate is 10 ml/min. The structures of the products were characterized by nuclear magnetic resonnance (NMR) spectroscopy and mass spectrometry (MS) (Bruker AVIII, 500 MHz). The purified products were vacuum evaporated to dryness, dissolved in methanol-*d4* or acetone-*d6*. 1D and 2D NMR spectra were recorded.

### UPLC and UPLC-MS/MS analyses of reaction products

The analyses of reaction products and determination of conversion rate were performed on an Agilent 1260 Infinity UPLC system (Agilent Technologies, Germany)^17^. The analyses were performed on an Agilent ZORBAX SB-C18 column (3.0 × 100 mm, 1.8 μm). The flow rate was 0.3 ml/min, the column temperature was set at 40 °C, and the injection volume was 10 μl. The chromatogram was monitored at 280 nm. The mobile phases comprised solvents A (ultrapure water) and B (methanol). The elution program was as follows: 0–30 min, 5–100% B; 30–40 min, 100% B^18^.

UPLC-MS/MS was analysed on a maXis LC-ESI-QTOF-MS system (Bruker, Germany) equipped with an Agilent ZORBAX SB-C18 column (3.0 × 100 mm, 1.8 μm). The elution program was the same as UPLC analysis. Ionization of the analytes was achieved by using electron spray ionization interface in negative mode. The collision voltage was 10 eV. Mass scan was set in the range of *m*/*z* 50–1000. The daughter ions were monitored at a collision voltage range of 28–42 eV^19^.

### Binding affinity prediction by molecular docking

The X-ray structure of estrogen receptor α and β in complex with agonist (2 R,3 S,4 R)-(4-hydroxyphenyl)-6-hydroxy-cyclopentyl[c]3,4-dihydro-2H-1- benzopyran (PDB id: 2i0j and 2i0g) were used as the templates^20^. Autodocktools (version 1.5.6rc3) was used to dock estrogen receptor α/β and the tested chemicals^21^. The protein was checked for any misssing atoms, removed water and added hydrogen. The ligand was drawed and saved as a mol2 file. It was opened in autodocktools and saved as PDBQT file. A grid was generated for protein with the ligand centered. Genetics algorithm was used for docking^22^. The binding affinity was recorded to evaluate the potential to be estrogen receptor agonist.

## Results and Discussion

### Synthesis of anhydroicaritin, icaritin and wushanicaritin

#### Enzyme hydrolysis of icariin

**Figure 1:**
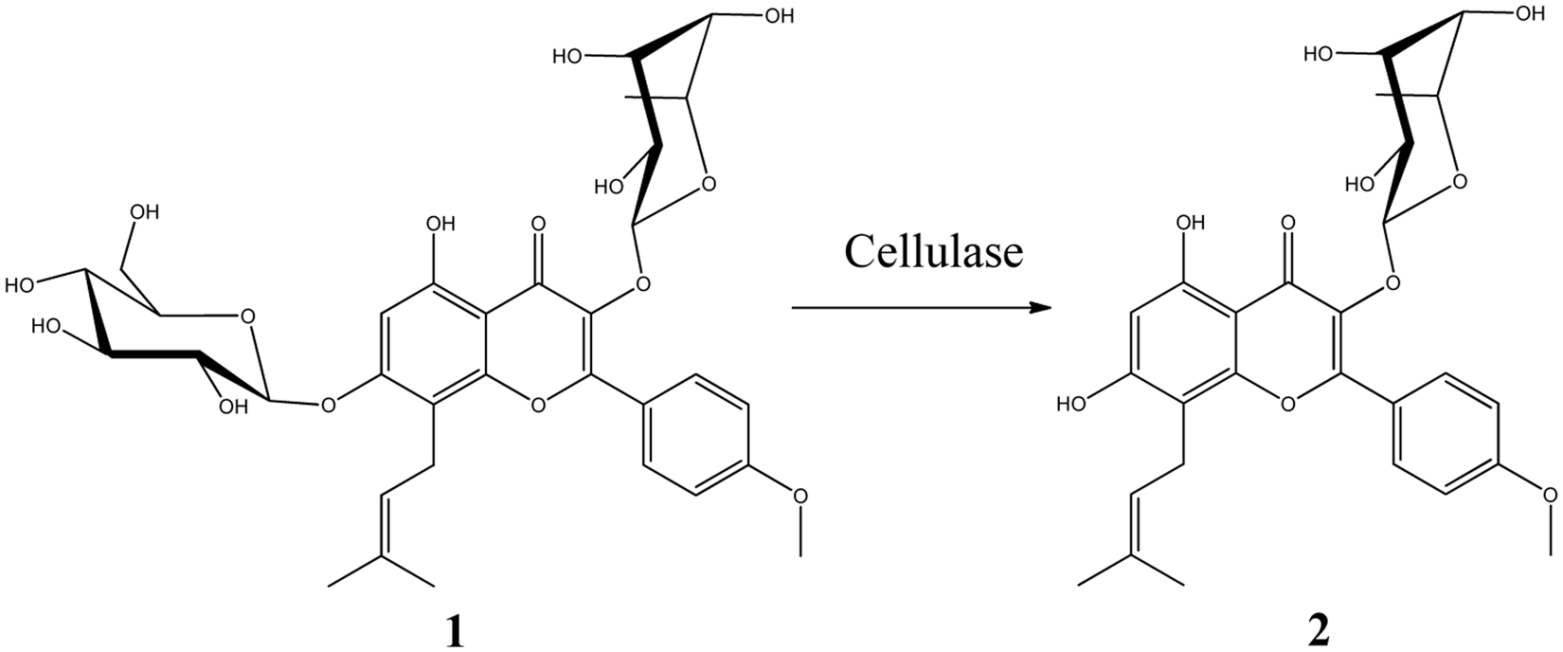
The reaction product of icariin hydrolysed by cellulase at 37 °C for 2.5 h. Yield of **2**, 94.8%.

**Figure 2:**
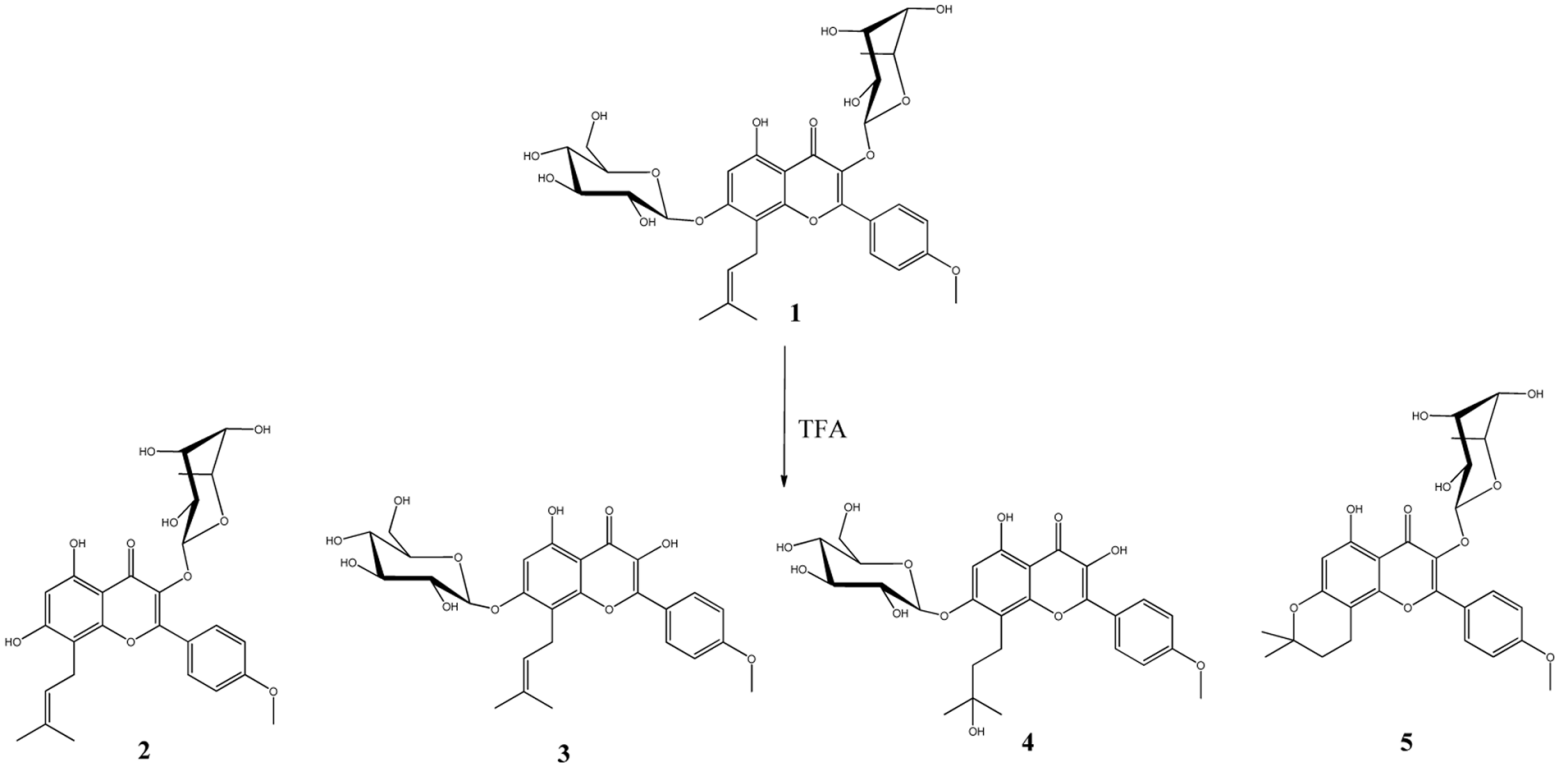
The reaction products of icariin hydrolysed by TFA at 60 °C for 1.0 h. Yields of **2**, 3.5%; **3**, 49.3%; **4**, 32.7%; **5**, 1.6%.

Three enzymes, including cellulase, β-glucosidase and β-dextranase, were used to hydrolyse icariin, respectively. There was only one product generated after enzyme hydrolysis (Fig. 1). It was purified and subjected to NMR analysis. The ^1^H and ^13^C chemical shifts of this chemical are listed in Table 1S and Fig. 2S. When comparing with icariin (**1**, Fig. 1S), the missing of glucosyl signals indicated the loss of this moiety. An upfield shift of H-6 was observed at 6.26 ppm. The chemical shifts of C-7 and C-8 were changed accordingly. The rhamnosyl with anomeric signals (5.40/103.7 ppm) was detected^23^. It confirmed that these enzymes could not degrade this unit. The above information confirmed the presence of **baohuoside I** (**2**).

UPLC-QTOF-MS is used to determine the precise molecular weight and fragmentation pattern of baohuoside I in negative mode (Table 2S). A parent ion [M-H]^−^ at *m*/*z* 513.1761 was observed. *m*/*z* 367.1112 indicated the loss of rhamnosyl. Two peaks at *m*/*z* 323.0921 and 311.0573 suggested the cleavage of prenyl and loss of C_3_H_6_ and C_4_H_8_ moieties, respectively^24^.

Though β-glucosidase has been reported to be effective in hydrolysis of icariin^25^, its effectiveness was much lower than cellulase in this work (Fig. 10S). The icariside I yield reached 94.8% by cellulase hydrolysis, while the yield was lower than 50% when β-glucosidase or β-dextranase was used. Temperature influenced the glucosyl cleavage to a certain extent. The highest yield of icariside I was obtained when 37 or 60 °C were applied. It indicated a broad temperature tolerance of cellulase. Out of this range made the hydrolysis efficiency decreased sharply. Hydrolysis time showed a weak effect on icariside I yield. 2.5 h was the optimal value. Further extension of reaction time led to a slight decrease of icariside I, which might be due to the degradation.

#### Acid hydrolysis of icariin

**Figure 3:**
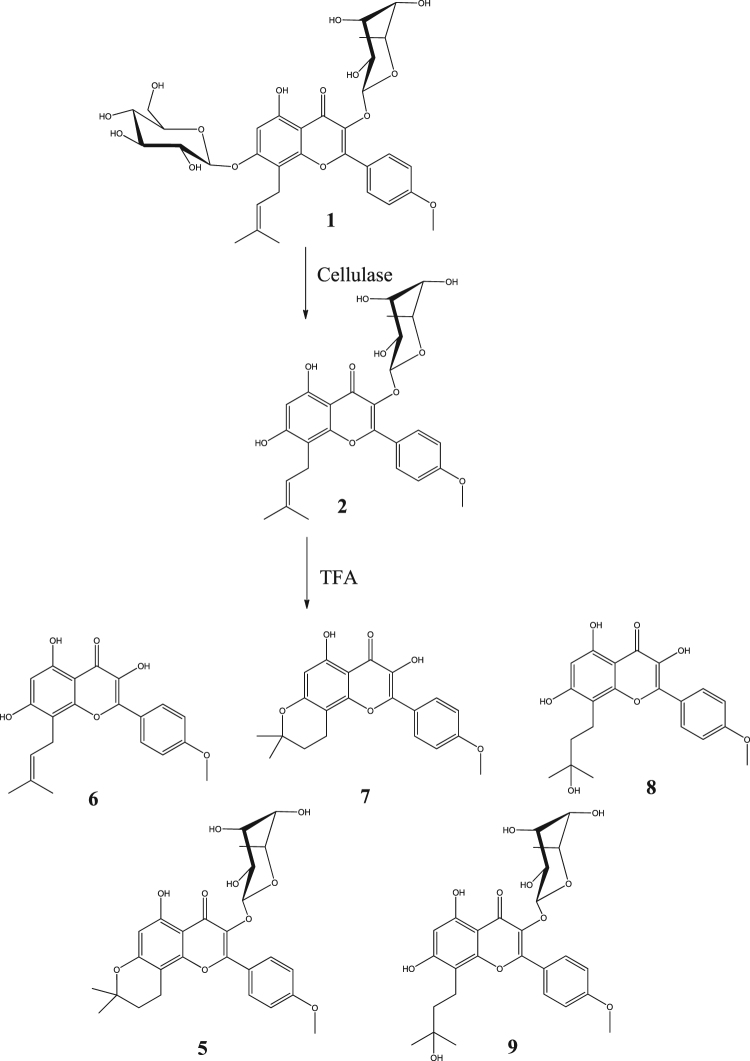
The reaction products of icariin hydrolysed by cellulase firstly and then TFA. Yields of **5**, 10.5%; **6**, 61.1%; **7**, 12.2%; **8**, 3.7%; **9**, 7.6%.

**Figure 4:**
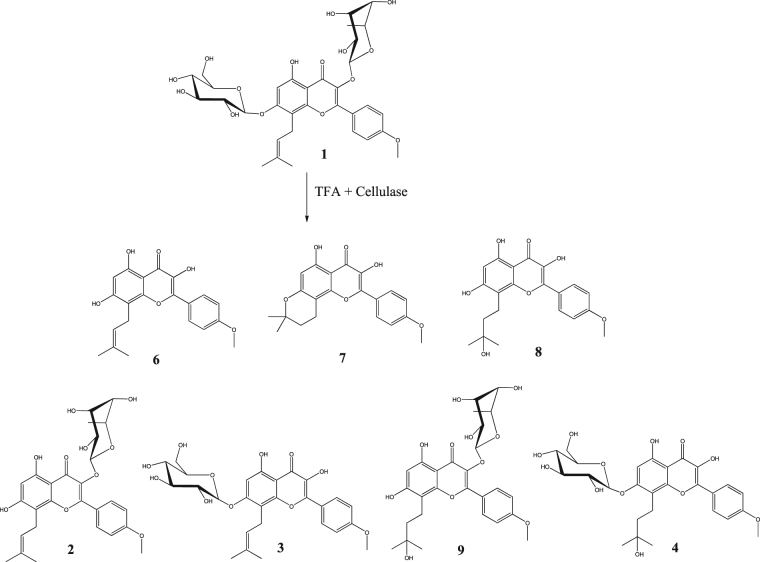
The reaction products of icariin hydrolysed by TFA firstly and then cellulase. Yields of **2**, 10.5%; **3**, 7.3%; **4**, 4.4%; **6**, 28.8%; **7**, 16.9%; **8**, 25.3%; **9**, 2.1%.

**Figure 5:**
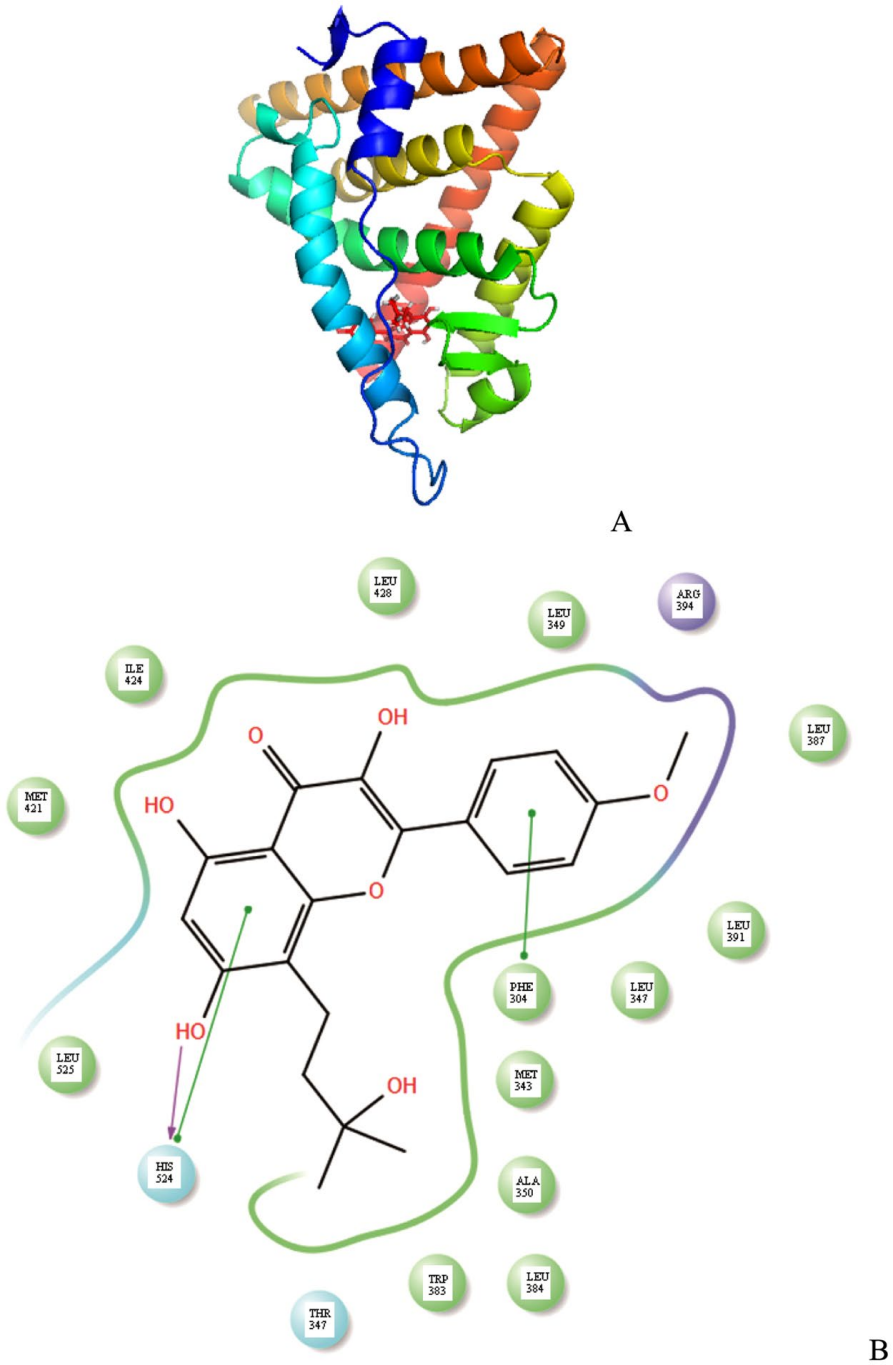
Docking simulation of estrogen receptor α in complex with wushanicaritin. (**A**) The steric structure of complex; (**B**) The interactions between amino acids and wushanicaritin.

Icariin was hydrolysed by TFA and four products were detected (Fig. 2). They were purified by C-18 column and identified by UPLC-MS/MS and NMR. Please see Table 2S and Figs 3S–5S. Chemical **3** showed a parent ion [M-H]^−^ at *m*/*z* 529.1765, which was equal to the molecular weight of icariside I^26^. The fragments also confirmed this identificaiton. A fragment at *m*/*z* 473.1316 suggested the breakage of prenyl between C-1 and C-2. The loss of glucosyl was observed by the peak at *m*/*z* 367.1115. The anomeric signals of rhamnosyl (5.40/103.7 ppm) were not detected. It indicated the removal of rhamnosyl from icariin. By comparison with NMR signals of icariin, **3** was identified as icariside I.

Chemical **4** was analysed to have a parent ion [M-H]^−^ at *m*/*z* 547.1892. It was 18 larger than icariside I (**3**), which implicated the presence of H_2_O. In NMR spectra, a quaternary carbon signal C-3″ at 71.7 ppm and C-2″ signal at 18.8 ppm further indicated the hydration of prenyl. Through comparing with NMR signals of icariside I and literatures^27^, chemical **4** was identified as maohuoside A.

A minor amount of chemical **5** was detected. It had a parent ion [M-H]^−^ at *m*/*z* 513.1759. The fragment ion at *m*/*z* 367.1115 indicated the loss of rhamnosyl. The anomeric signals of 5.44/102.2 ppm in NMR spectra also confirmed the presence of rhamnosyl. A cyclization between C-3″ of prenyl and 6-OH was observed by the NMR signals 2.84/16.8 (H-1″/C-1″), 1.88/32.1 (H-2″/C-2″), 77.5 (C-3″), 1.36/26.4 (H-4″/C-4″ and H-5″/C-5″). Therefore, chemical **5** was identified as anhydroicaritin 3-*O*-rhamnoside.

The four chemicals generated by TFA hydrolysis of icariin were compared. **2** and **5** contained rhamnosyl, but no glucosyl, while **3** and **4** contained glucosyl but no rhamnosyl. Rhamnosyl was easier to be cleaved than glucosyl by acid. In acidic conditions, the prenyl was unstable and readily to be hydrated or cyclized. No products without both rhamnosyl and glucosyl were detected. It indicated that TFA hydrolysis could not directly generate icaritin. Therefore, combination of cellulase and TFA hydrolysis was carried out in the following work.

### Combination of cellulase and acid hydrolysis

**Figure 6:**
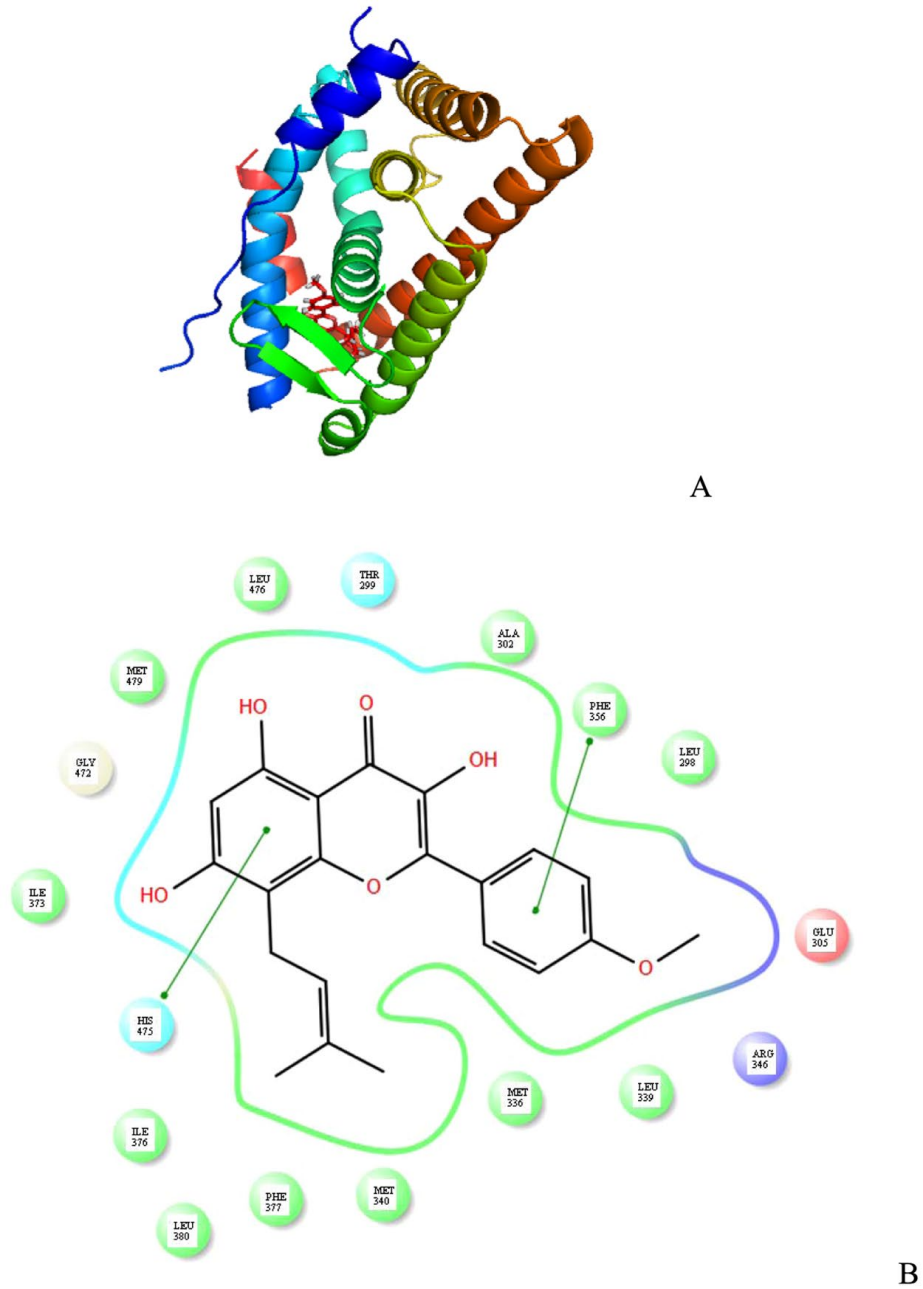
Docking simulation of estrogen receptor β in complex with icaritin. (**A**) The steric structure of complex; (**B**) The interactions between amino acids and icaritin.

When icariin was hydrolysed by cellulase firstly and then by TFA, five main products were detected (Figure 11S). The reaction route and products are shown in Fig. 3. Chemical **6** was the dominant product which had a parent ion [M-H]^−^ at *m*/*z* 367.1142. No rhamnosyl and glucosyl signals were observed in the NMR signals (Fig. 6S). Their losses led to the upfield movements of H-6, C-2, C-4 and downfield shift of C-3. The fragment ion at *m*/*z* 352.0974 was due to the loss of methyl. *m*/*z* 311.0601 was produced by the cleavage of prenyl between C-1″ and C-2″. These information confirmed that chemical **6** was icaritin^10^.

Chemical **7** had a parent ion [M-H]^−^ at *m*/*z* 367.1135. It was consistent to the parent ion of icaritin. By comparing the NMR signals (Figure 7S), the double bond of prenyl was not found in chemical **7**. The quaternary carbon signal of 77.7 ppm at C-3″ and two-proton signal of 1.95 ppm at H-2″ suggested a cyclization between C-3″ and 7-OH. Therefore, chemical **7** was identified as anhydroicaritin.

Chemical **8** had a parent ion [M-H]^−^ at *m*/*z* 385.1165. It was 18 more than that of icaritin. The quaternary carbon signal of 71.7 ppm at C-3″ and 2 H signals of 2.90–2.95 ppm at H-2″ suggested the hydation of icaritin (Table 1S and Figure 8S). It was called wushanicaritin^28^. Chemical **9** was identified to be 3-O-rhamnosyl wushanicaritin by the parent ion [M-H]^−^ at *m*/*z* 531.1246 and NMR signals (Figure 9S).

When icariin was hydrolysed by TFA firstly and then cellulase, seven chemicals were produced as the main reaction products (Fig. 4, Figure 12S). These chemicals were identified as baohuoside I (**2**), icariside I (**3**), maohuoside A (**4**), icaritin (**6**), anhydroicaritin (**7**), wushanicaritin (**8**) and wushanicaritin 3-*O*-rhamnoside (**9**). Anhydroicaritin 3-*O*-rhamnoside (**5**) was not detected as a product. The yields of icaritin, anhydroicaritin and wushanicaritin were calcalated to be 28.8%, 16.9% and 25.3%, respectively.

When icariin was hydrolysed by cellulase firstly and then TFA, baohuoside I (**2**) was not detected in the products. It was cyclized into anhydroicaritin 3-*O*-rhamnoside (**5**). The yields of icaritin, anhydroicaritin and wushanicaritin were calcalated to be 61.1%, 12.2% and 3.7%, respectively. Therefore, hydrolysis by cellulase firstly and then TFA would be a good choice for icaritin preparation.

### Docking study

Molecular docking simulation was performed for icaritin, anhydroicaritin and wushanicaritin, in order to predict the estrogen receptor α/β-agonist interactions. The most favorable docking conformation was retrieved from calculation. As shown in Table 3S, in the docking model, wushanicaritin showed high binding affinities to estrogen receptors α (−8.48 kcal/mol) and β (−7.97 kcal/mol). Icaritin only exhibited a good binding affinity to estrogen receptor β (−9.46 kcal/mol). Anhydroicaritin showed poor binding affinities to both estrogen receptors. Icaritin was predicted to have a good selectivity due to its specific binding affinity to estrogen receptor β.

Figure 5 shows the docking conformation of estrogen receptor α and wushanicaritin. The ligand binding domain of estrogen receptor α comprises of three anti-parrallel α-helices layers, including a central layer and two outside layers^29^. The residues Met343, Leu346, Leu349, Ala350, Trp383, Leu384, Leu391, Leu387, Met421, Ile424, Leu428 and Leu525 are predicted to be involved in the positioning of wushanicaritin by hydrophobic forces. Phe404 was predicted to interact with wushanicaritin by π-π stacking. His524 was predicted to generate a hydrogen bond and π-π stacking interaction to stabilize wushanicaritin. The conformation of H12 in the complex determines the binding possibility of coactivator to activate function domain (AF-2). In the estrogen receptor α/wushanicaritin complex, H12 was predicted to seal the ligand-binding cavity and to generate a competent AF-2 that could interact with coactivator. This could be helpful to explain how wushanicaritin acted as an agonist.

Figure 6 shows thne predicted conformation of estrogen receptor β in complex with icaritin. Estrogen receptor β is present similiarly to estrogen receptor α as a sandwich made by a central layer and two outside layers. H12 forms a lid on the binding cavity. π-π stacking interaction was observed by residues His475 and Phe356. No hydrogen bonds were detected to position icaritin in the predicted conformation. Hydrophobic forces from Leu298, Ala302, Leu476, Met479, Ile373, Ile376, Leu380, Phe377, Met340, Met336 and Leu339 were predicted to be involved in stabilizing icaritin.

## Conclusions

A combination of cellulase and TFA hydrolysis was confirmed to be an efficient technique to synthesize icaritin, wushanicaritin and anhydroicaritin. This technique extended the application of cellulase in icariside preparation and made production of three aglycones in one pot possible^30^. It was easy to carry out and to produce at a large scale when comparing synthesis from kaempferol^31^. Icaritin, wushanicaritin and anhydroicaritin are good drug candidates due to their good performance against cancer and osteoporosis. Developing a cost effective technqiue is of great interest for medicine industry. As the prenylation pattern produced a significant effect on the activity performance, it is required to conduct more bioactivity evaluations, including the estrogen receptor modulatory mechanisms *in vitro* and *in vivo*, to understand the potents of these chemicals in medicines. Some bioactivites, like anti-breast cancer, anti-osteoporosis or memory improving capabilities, should be evaluated, as estrogen receptor modulatory behaviours are involved in the mechanism of action.

## Electronic supplementary material

supplementary information without marked changes
