# Supplementary material for: Synthesis of prenylated flavonols and their potents as estrogen receptor modulator
Source: Sci Rep. 2017 Sep 29;7:12445. doi: 10.1038/s41598-017-12640-9 (PMC5622168; doi:10.1038/s41598-017-12640-9)
Supplement: Supplementary file 1 — supplementary information without marked changes [file 41598_2017_12640_MOESM1_ESM.pdf]

Supplementary information

Synthesis of prenylated flavonols and their potents as estrogen receptor modulator

Zhenru Tao, Juan Liu, Yueming Jiang, Liang Gong, Bao Yang

Table 1S. NMR assignments of **1-9**.

	**1 (Methanol-*d4*)**			**2 (Methanol-*d4*)**	
	^{1}H	^{13}C		^{1}H	^{13}C
2		155.2	2		155.2
3		136.7	3		136.8
4		180.4	4		180.4
4a		107.8	4a		107.9
5		162.3	5		162.2
6	6.66,s	99.5	6	6.26,s	98.8
7		163.8	7		162.1
8		110.8	8		107.4
8a		159.6	8a		159.8
1’		124.2	1’		124.2
2’	7.90,d,9.0	132.1	2’	7.87,d,9.0	132.1
3’	7.11,d,9.0	115.3	3’	7.10,d.9.0	115.5
4’		161.3	4’		161.3
5’	7.11,d,9.0	115.3	5’	7.10,d.9.0	115.5
6’	7.90,d,9.0	132.1	6’	7.87,d,9.0	132.1
CH_3O-	3.90,s	56.2		3.89,s	56.2
1’’	3.55-3.60, 3.61-3.66,m	22.9	1’’	3.54-3.59, 3.60-3.65,m	22.9
2’’	5.20,t,7.0	123.6	2’’	5.20,t,7.0	123.7
3’’		132.9	3’’		132.8
4’’	1.64,s	26.0	4’’	1.66,s	26.0
5’’	1.72,s	18.6	5’’	1.72,s	18.6
Rhamnosyl					
1	5.42,d,1.5	103.7	1	5.40,d,1.5	103.7
2	4.22,dd,1.5,3.5	72.1	2	4.22,dd,1.5,3.5	72.1
3	3.70,dd,3.5,9.0	71.9	3	3.70,dd,3.5,9.0	71.9
4	3.31-3.37,m	73.4	4	3.46-3.52,m	73.4
5	3.27-3.33,m	72.1	5	3.41-3.47,m	72.1
6	0.90,d,6.0	17.8	6	0.90,d,6.0	17.8
Glucosyl					
1	5.07,d,7.5	102.0			
2	3.51-3.59,m	75.0			
3	3.48-3.56,m	77.4			
4	3.43,d,9.5	71.3			
5	3.48-3.56,m	78.5			
6	3.74,dd,5.5,12.5<br>3.90,dd,2.0,12.5	62.4			

Table 1S-continued

	3 (Acetone-*d6*)			4 (Methanol-*d4*)	
	^{1}H	^{13}C		^{1}H	^{13}C
2		146.2	2		148.1
3		137.0	3		137.8
4		177.2	4		178.1
4a		104.3	4a		106.2
5		162.3	5		162.0
6	6.66,s	99.5	6	6.62,s	99.0
7		163.8	7		162.8
8		110.7	8		111.4
8a		159.4	8a		154.7
1’		124.2	1’		125.2
2’	8.26,d,9.0	130.2	2’	8.23,d,9.0	130.8
3’	7.14,d,9.0	115.3	3’	7.03,d,9.0	115.1
4’		162.3	4’		160.4
5’	7.14,d,9.0	115.3	5’	7.03,d,9.0	115.1
6’	8.26,d,9.0	130.2	6’	8.23,d,9.0	130.8
CH_3O-	3.91,s	56.3		3.87,s	54.5
1’’	3.56-3.61, 3.62-3.67,m	22.7	1’’	1.68-1.73,m	44.0
2’’	5.30,t,7.0	123.6	2’’	2.92-2.98,m	18.8
3’’		132.9	3’’		71.7
4’’	1.67,s	26.0	4’’	1.32,s	29.1
5’’	1.84,s	18.6	5’’	1.32,s	29.1
Glucosyl					
1	5.07,d,7.5Hz	102.2	1	5.02,d,7.0	102.4
2	3.53-3.61,m	75.2	2	3.50-3.57,m	75.1
3	3.48-3.56,m	77.5	3	3.49-3.55,m	78.2
4	3.44,d,9.5	71.3	4	3.44-3.48,m	71.4
5	3.49-3.57,m	78.6	5	3.52-3.57,m	78.5
6	3.74,dd,5.5,12.5	62.6	6	3.73,dd,5.5,12.5	62.5
	3.90,dd,2.0,12.5			3.89,dd,2.0,12.5	

Table 1S-continued

	**5 (Methanol-*d4*)**			**6 (Acetone-*d6*)**	
	^{1}H	^{13}C		^{1}H	^{13}C
2		155.0	2		146.7
3		136.4	3		136.9
4		180.3	4		177.0
4a		104.5	4a		104.4
5		163.2	5		160.1
6	6.12,s	99.8	6	6.36,s	98.9
7		161.2	7		162.2
8		100.7	8		107.3
8a		154.7	8a		155.2
1’		125.1	1’		124.8
2’	7.90,d,9.0	115.3	2’	8.26,d,9.0	130.5
3’	7.09,d,9.0	130.7	3’	7.14,d,9.0	115.2
4’		160.5	4’		162.3
5’	7.09,d,9.0	130.7	5’	7.14,d,9.0	115.2
6’	7.90,d,9.0	115.3	6’	8.26,d,9.0	130.5
CH_3O-	3.89,s	56.1		3.91,s	56.1
1’’	2.84-2.88,m	16.8	1’’	3.57,d,7.0	22.6
2’’	1.88,t,7.0	32.1	2’’	5.28,t,7.0	123.6
3’’		77.5	3’’		132.3
4’’	1.36,s	26.4	4’’	1.67,s	26.1
5’’	1.36,s	26.4	5’’	1.83,s	18.3
Rhamnosyl					
1	5.44,d,1.5	102.2			
2	4.24,dd,1.5,3.5	72.1			
3	3.70,dd,3.5,9.0	71.9			
4	3.31-3.37,m	73.4			
5	3.27-3.33,m	72.1			
6	0.90,d,6.0	17.8			

Table 1S-continued

	7 (acetone-*d6*)			8 (acetone-*d6*)	
	^{1}H	^{13}C		^{1}H	^{13}C
2		146.6	2		147.6
3		137.3	3		137.5
4		176.6	4		177.9
4a		104.6	4a		104.8
5		159.6	5		163.2
6	6.14,s	99.9	6	6.24,s	99.0
7		161.2	7		162.7
8		100.9	8		108.5
8a		154.8	8a		155.6
1'		123.8	1'		125.5
2'	7.16,d,9.0	115.0	2'	7.05,d,9.0	115.0
3'	8.29,d,9.0	130.2	3'	8.26,d,9.0	130.8
4'		162.2	4'		160.3
5'	8.29,d,9.0	130.2	5'	8.26,d,9.0	130.8
6'	7.16,d,9.0	115.0	6'	7.05,d,9.0	115.0
CH_3O-	3.91,s	55.9		3.88,s	56.0
1''	2.96,t,7.0	16.8	1''	1.68-1.73,m	44.1
2''	1.95,t,7.0	32.3	2''	2.90-2.95,m	19.0
3''		77.7	3''		71.7
4''	1.39,s	26.8	4''	1.32,s	29.2
5''	1.39,s	26.8	5''	1.32,s	29.2

Table 1S-continued

	9 (methanol-*d4*)	
	^{1}H	^{13}C
2		155.2
3		136.7
4		180.4
4a		104.7
5		163.3
6	6.26,s	99.1
7		162.5
8		108.5
8a		155.8
1’		125.5
2’	7.93,d,9.0	115.2
3’	7.09,d,9.0	130.5
4’		160.5
5’	7.09,d,9.0	130.8
6’	7.93,d,9.0	115.0
CH_3O-	3.89,s	56.1
1’’	1.68-1.73,m	44.3
2’’	2.83-2.87,m	19.1
3’’		71.6
4’’	1.26,s	29.2
5’’	1.26,s	29.2
Rhamnosyl		
1	5.42,d,1.5	102.2
2	4.24,dd,1.5,3.5	72.1
3	3.72,dd,3.5,9.0	71.9
4	3.34-3.40,m	73.4
5	3.30-3.36,m	72.1
6	0.90,d,6.0	17.8

Table 2S. Mass fragments of **1-9** determined by UPLC-MS/MS in negative mode [a]

Chemicals	Parent ions (*m*/*z*)	Fragment ions (*m*/*z*)
**1**	675.1053	529.0571 (Rha loss), 513.1215 (Glc loss), 367.0812 (loss of Rha and Glc)
**2**	513.1761	367.1112 (Rha loss), 323.0921 (prenyl cleavage and Rha loss), 311.0573 (prenyl cleavage and Rha loss)
**3**	529.1765	473.1316 (prenyl cleavage), 367.1115 (Glc loss), 311.0570 (prenyl cleavage and Glc loss)
**4**	547.1892	385.1332 (Glc loss), 367.1223 (losses of Glc and hydroxyl)
**5**	513.1759	367.1115 (Rha loss), 352.0932 (losses of Rha and methyl)
**6**	367.1142	352.0974 (methyl loss), 325.1082 (prenyl cleavage), 311.0601 (prenyl cleavage)
**7**	367.1135	352.0974 (methyl loss), 311.0573 (prenyl cleavage)
**8**	385.1165	367.1026 (hydroxyl loss), 370.0962 (methyl cleavage)
**9**	531.1246	385.0791 (Rha loss), 370.0962 (losses of Rha and methyl)

[a] Rha, rhamnosyl; Glc, glucosyl.

Table 3S. The binding affinity (kcal/mol) of anhydroicaritin, icaritin and wushanicaritin to both estrogen receptors in docking study.

Chemicals	Estrogen receptor α	Estrogen receptor β
Anhydroicaritin	-5.11	-5.37
Icaritin	-5.90	-9.46
Wushanicaritin	-8.48	-7.97

Figure captions

Figure 1S. ^{1}H NMR spectra of icariin (**1**).

Figure 2S. ^{1}H NMR spectra of baohuoside I (**2**).

Figure 3S. ^{1}H NMR spectra of icariside I (**3**).

Figure 4S. ^{1}H NMR spectra of maohuoside A (**4**).

Figure 5S. ^{1}H NMR spectra of anhydroicaritin 3-*O*-rhamnoside (**5)**.

Figure 6S. ^{1}H NMR spectra of icaritin (**6**).

Figure 7S. ^{1}H NMR spectra of anhydroicaritin (**7**).

Figure 8S. ^{1}H NMR spectra of wushanicaritin (**8**).

Figure 9S. ^{1}H NMR spectra of wushanicaritin 3-*O*-rhamnoside (**9)**.

Figure 10S. Effects of enzymatic hydrolysis conditions on the icariside I yield. A, effects of three enzymes; B, effect of temperature when cellulase was used; C, effect of time when cellulase was used.

Figure 11S. The UPLC chromatogram of the reaction products when icariin was hydrolysed by cellulase firstly and then TFA.

Figure 12S. The UPLC chromatogram of the reaction products when icariin was hydrolysed by TFA firstly and then cellulase.

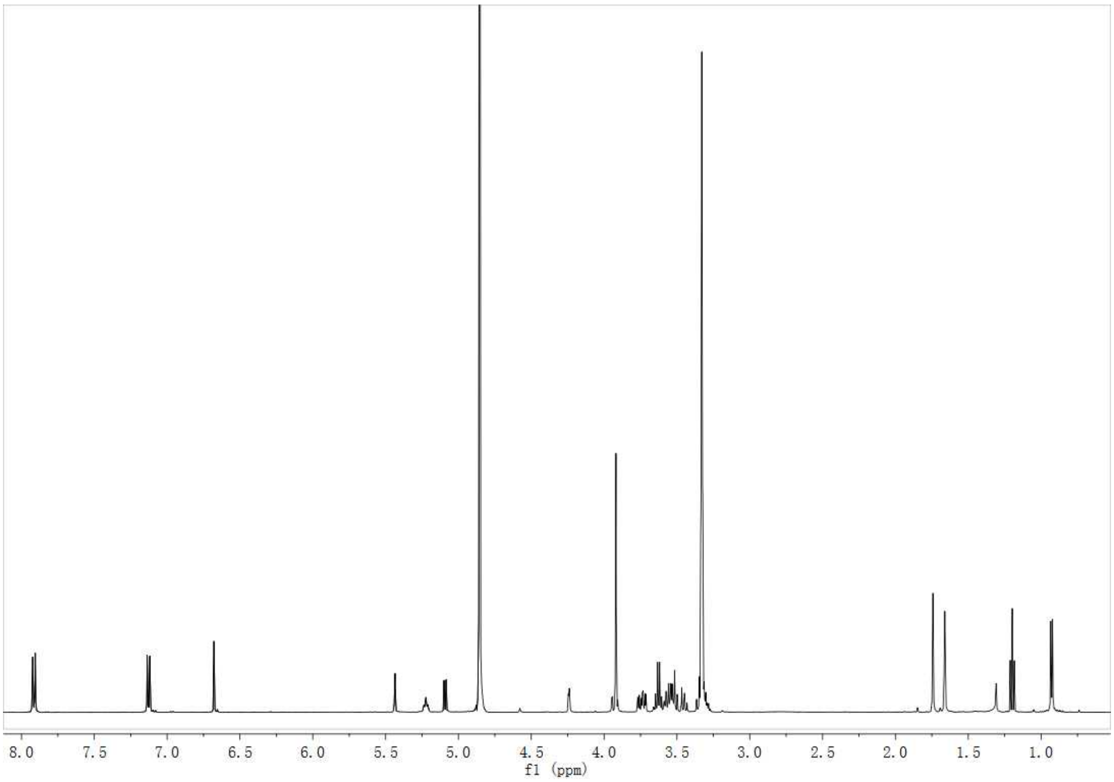

Figure 1S. ^{1}H NMR spectra of icariin (**1**).

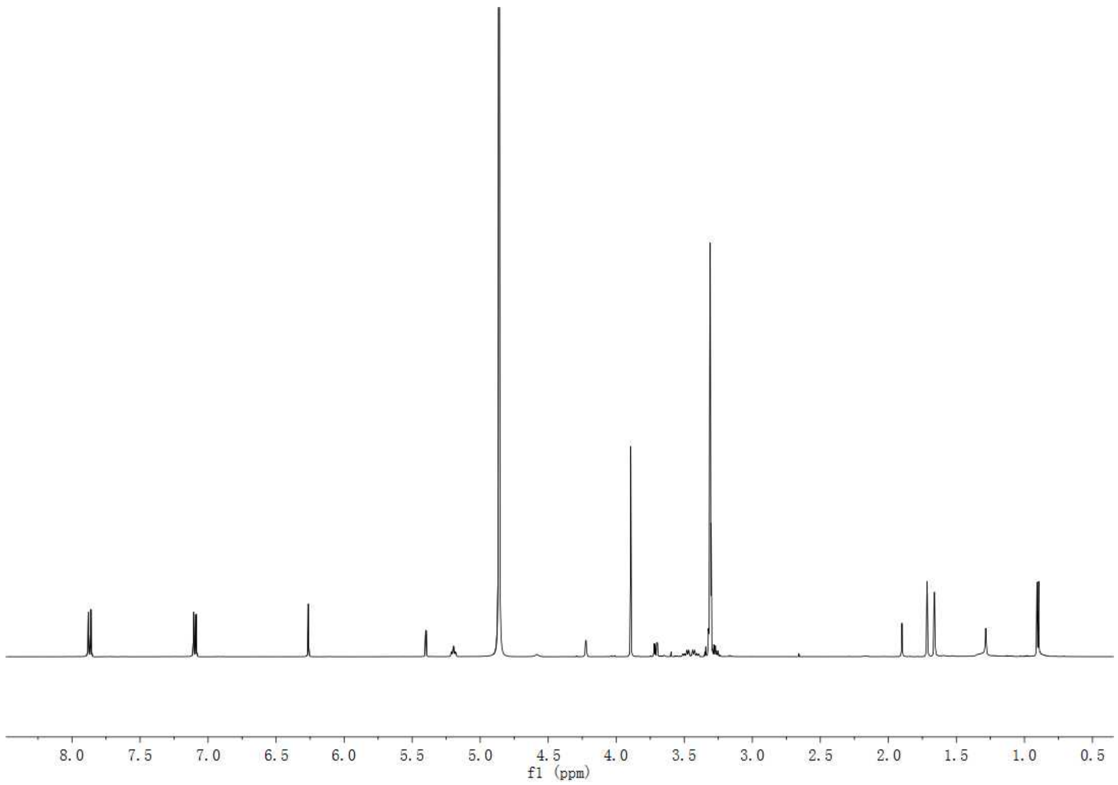

Figure 2S. ^{1}H NMR spectra of baohuoside I (**2**).

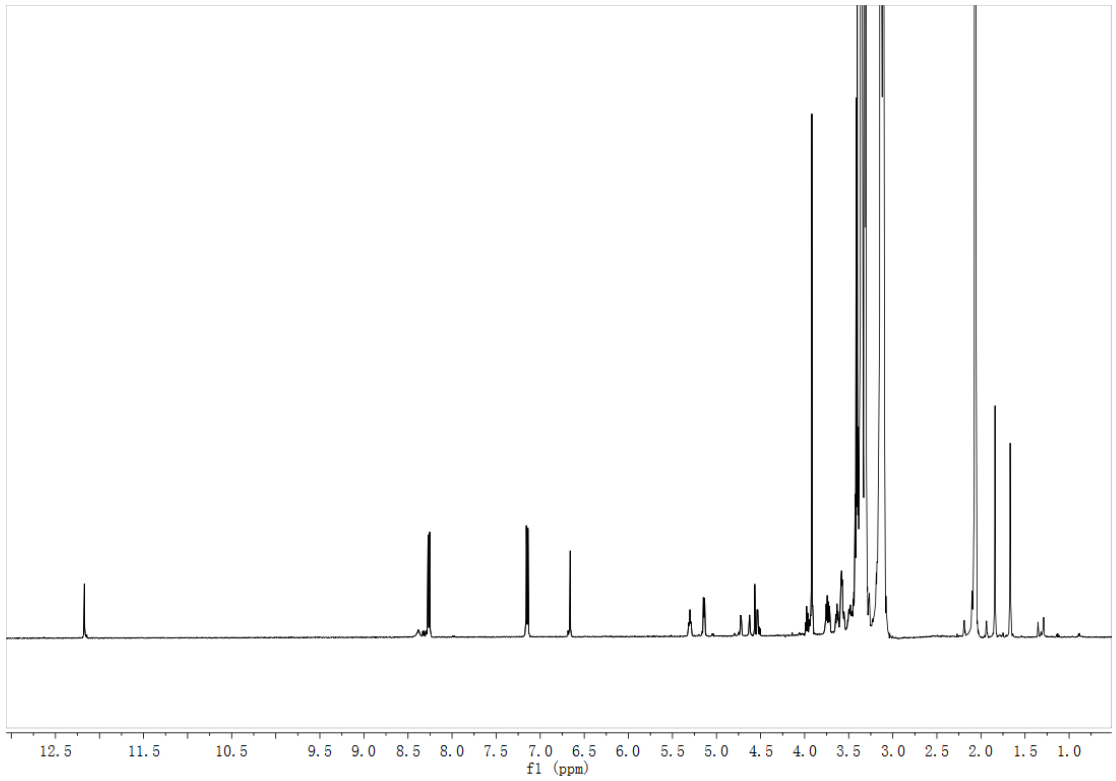

Figure 3S. ^{1}H NMR spectra of icariside I (**3**).

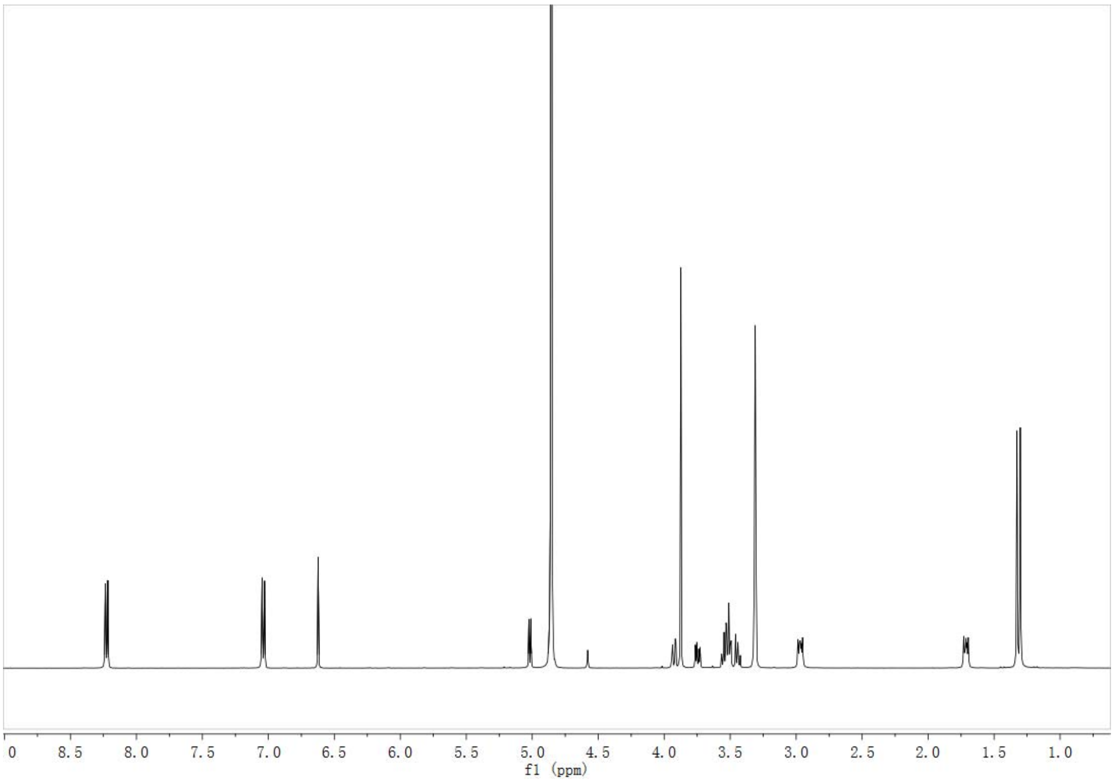

Figure 4S. ^{1}H NMR spectra of maohuoside A (4).

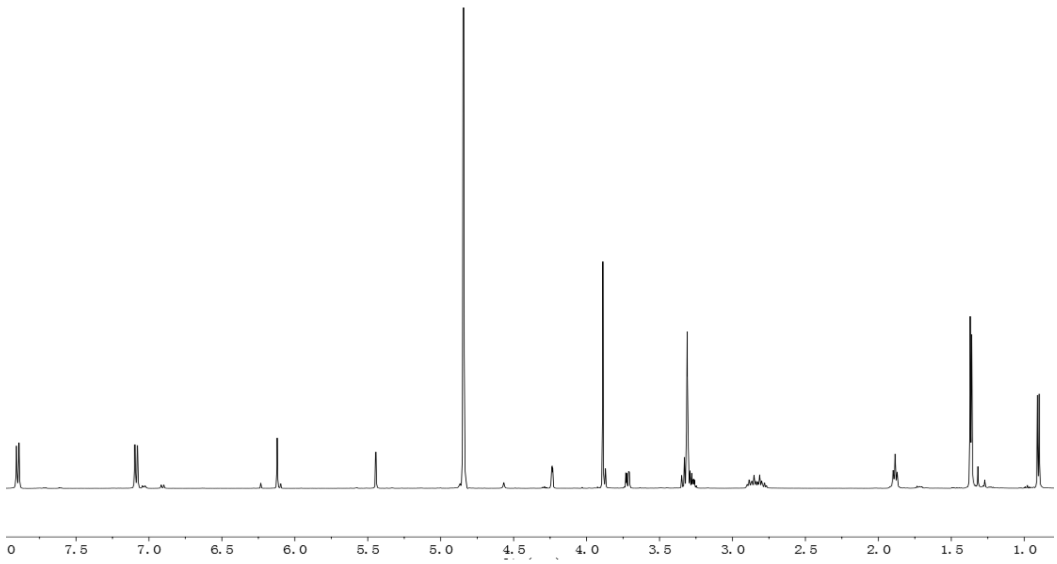

Figure 5S. ^{1}H NMR spectra of anhydroicaritin (**5**).

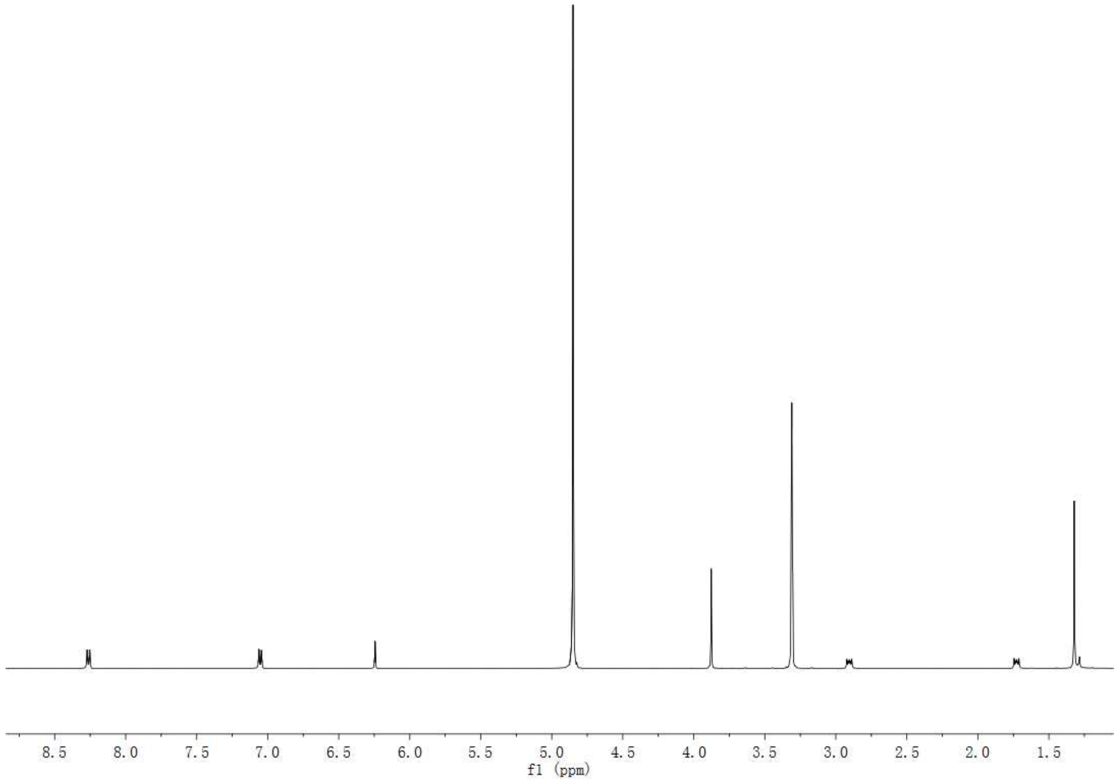

Figure 6S. ^1H NMR spectra of icaritin (**6**).

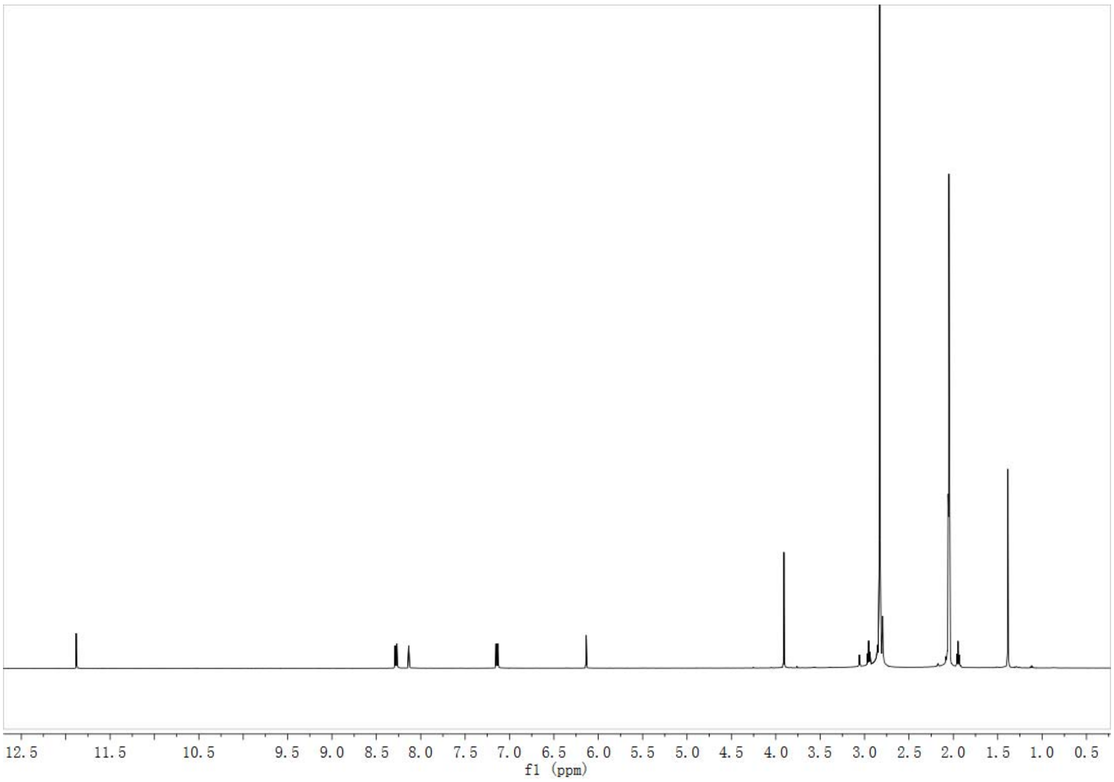

Figure 7S. ^{1}H NMR spectra of anhydroicaritin (**7**).

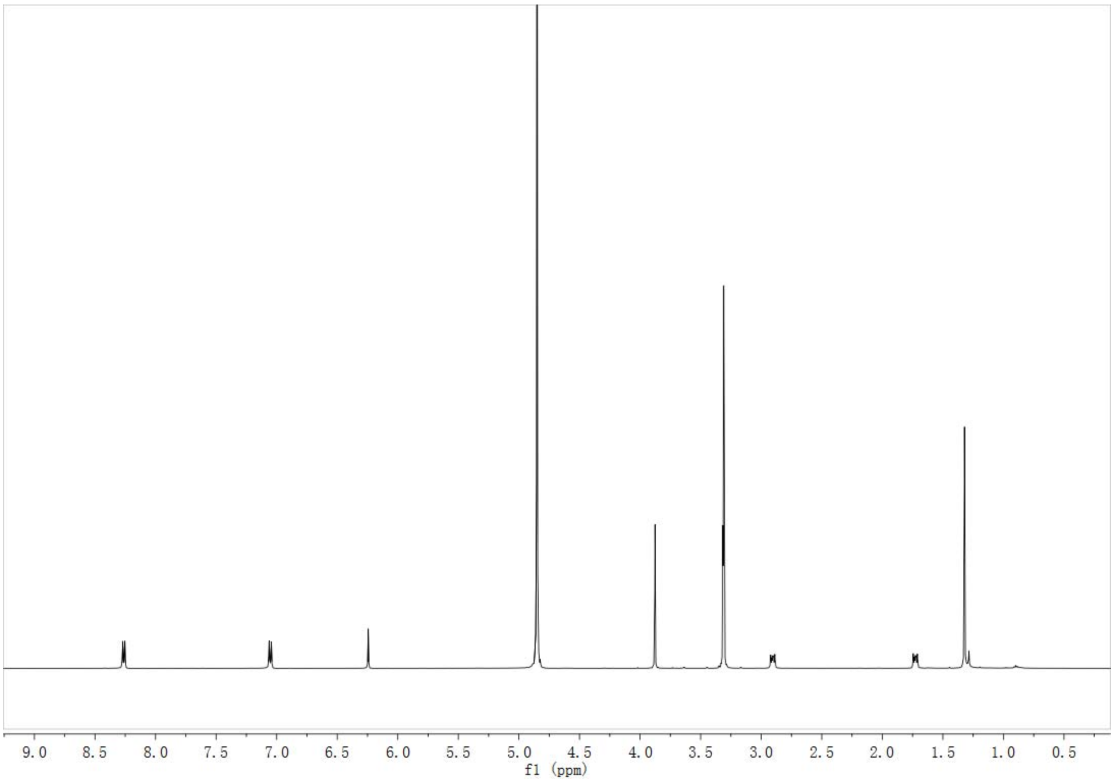

Figure 8S. ^{1}H NMR spectra of wushanicaritin (**8**).

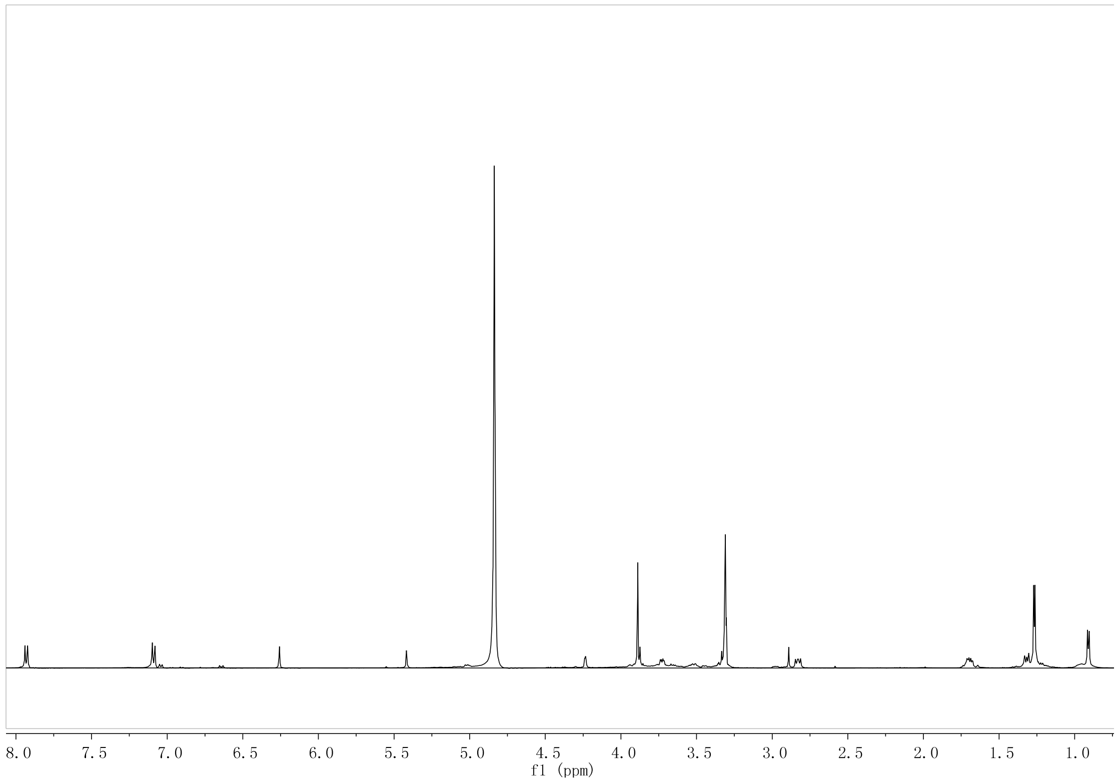

Figure 9S. ^{1}H NMR spectra of wushanicaritin (**9**).

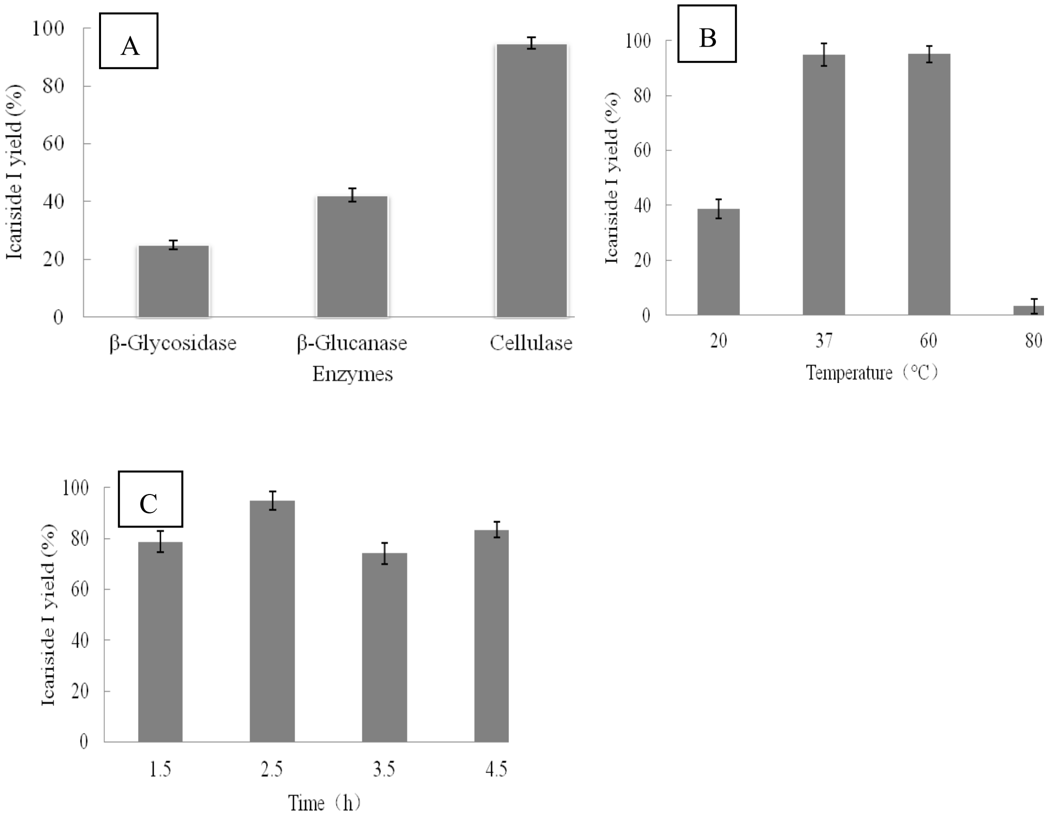

Figure 10S. Effects of enzymatic hydrolysis conditions on the icariside I yield. A, effects of three enzymes; B, effect of temperature when cellulase was used; C, effect of time when cellulase was used.

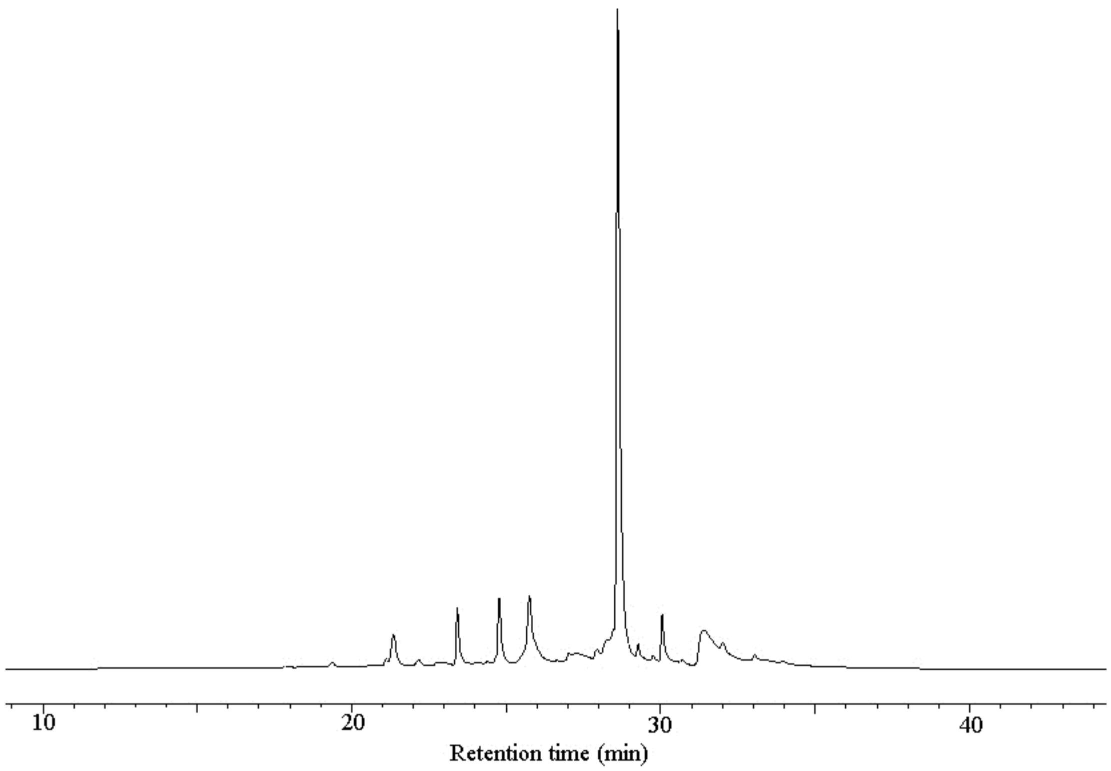

Figure 11S. The UPLC chromatogram of the reaction products when icariin was hydrolysed by cellulase firstly and then TFA.

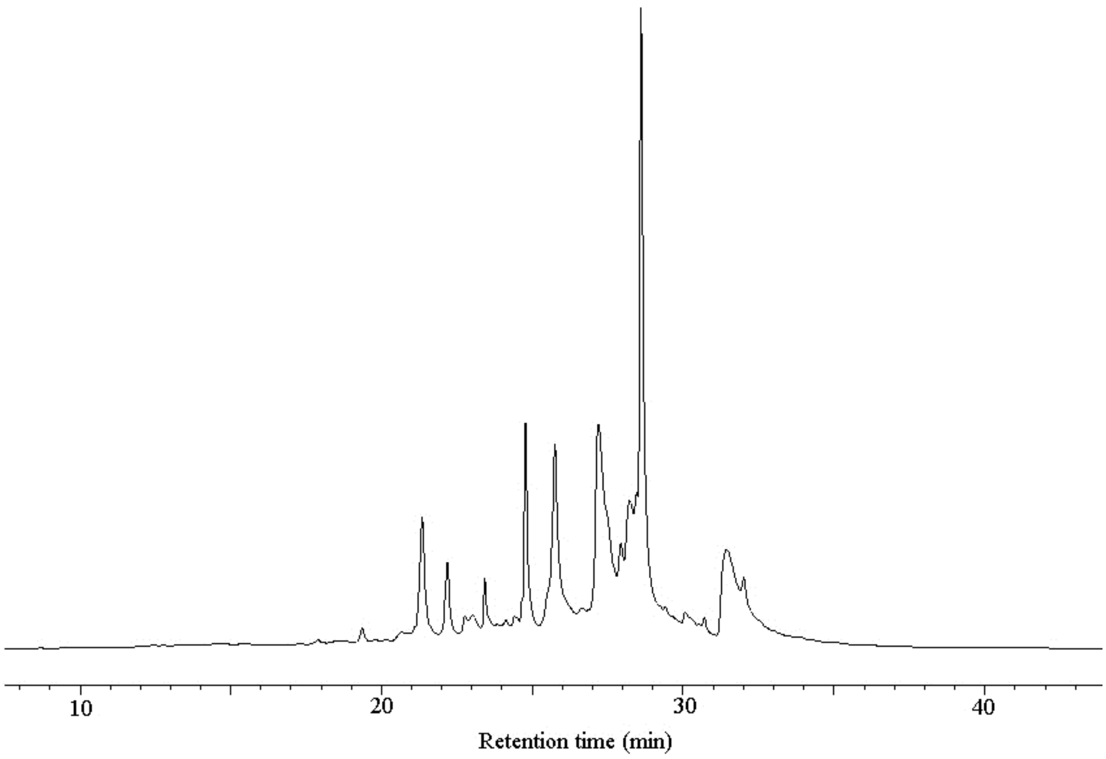

Figure 12S. The UPLC chromatogram of the reaction products when icariin was hydrolysed by TFA firstly and then cellulase.
